# COVID-19 Detection in CT/X-ray Imagery Using Vision Transformers

**DOI:** 10.3390/jpm12020310

**Published:** 2022-02-18

**Authors:** Mohamad Mahmoud Al Rahhal, Yakoub Bazi, Rami M. Jomaa, Ahmad AlShibli, Naif Alajlan, Mohamed Lamine Mekhalfi, Farid Melgani

**Affiliations:** 1 Applied Computer Science Department, College of Applied Computer Science, King Saud University, Riyadh 11543, Saudi Arabia; mmalrahhal@ksu.edu.sa; 2 Computer Engineering Department, College of Computer and Information Sciences, King Saud University, Riyadh 11543, Saudi Arabia; najlan@ksu.edu.sa; 3 Computer Science Department, College of Computer and Cyber Sciences, University of Prince Mugrin, Medina 42241, Saudi Arabia; r.jomaa@upm.edu.sa; 4 Computer Science Department, College of Computer and Information Sciences, King Saud University, Riyadh 11543, Saudi Arabia; alshibli@ksu.edu.sa (A.A.); mohamed.mekhalfi@alumni.unitn.it (M.L.M.); 5 Department of Information Engineering and Computer Science, University of Trento, 38123 Trento, Italy; melgani@disi.unitn.it

**Keywords:** COVID-19, vision transformer, computed tomography, X-ray images, deep learning

## Abstract

The steady spread of the 2019 Coronavirus disease has brought about human and economic losses, imposing a new lifestyle across the world. On this point, medical imaging tests such as computed tomography (CT) and X-ray have demonstrated a sound screening potential. Deep learning methodologies have evidenced superior image analysis capabilities with respect to prior handcrafted counterparts. In this paper, we propose a novel deep learning framework for Coronavirus detection using CT and X-ray images. In particular, a Vision Transformer architecture is adopted as a backbone in the proposed network, in which a Siamese encoder is utilized. The latter is composed of two branches: one for processing the original image and another for processing an augmented view of the original image. The input images are divided into patches and fed through the encoder. The proposed framework is evaluated on public CT and X-ray datasets. The proposed system confirms its superiority over state-of-the-art methods on CT and X-ray data in terms of accuracy, precision, recall, specificity, and F1 score. Furthermore, the proposed system also exhibits good robustness when a small portion of training data is allocated.

## 1. Introduction

Over the past two years, the world has endured an unprecedented pandemic, namely COVID-19, which is caused by Severe Acute Respiratory Syndrome Coronavirus 2 (SARS-CoV-2). Notwithstanding the tremendous efforts that have been undertaken to contain this pandemic at the global level, the world is still dealing with the heavy aftermath, ranging from human losses to economic recessions.

Common medical diagnostic methods of COVID-19 include antibody testing [1] and quantitative reverse transcription-polymerase chain reaction (qRT-PCR) [2,3]. The antibody-testing technique is typically fast, and results can be achieved in quasi real-time. However, its precision remains questionable as it may present high false negative rates for early and active infections. RT-PCR, on the other hand, is relatively much more accurate. However, its prolonged process does not qualify it for real-time use. Furthermore, it may not be as effective in discerning the presence of the virus if there is not enough traces of the virus in the body of the subject [3,4,5].

In this regard, both diagnostic methods are heavily dependent on human expertise to collect and analyze the samples. Moreover, hospitals and medical facilities in many countries have fallen short in their availability of test kits and in their ability to respond to the ongoing influx of test demands, which may encourage the spread of the virus. Thus, improved medical image analysis, if properly addressed, is believed to provide an auxiliary aid to medical experts.

Medical image analysis as a field of study has been gaining ground over the past decade on account of its (typically) non-invasive, quick, and automatic nature. Medical data constitute a paramount component in this sense. It may take the form of unidimensional/bidimensional signals [6,7], an image stack [8], or a large amount of data [9]. Furthermore, multimodal data sources can also be leveraged [10].

For the detection of COVID-19, X-ray [11,12], ultrasound [13,14], and Computed Tomography (CT) [15] represent the most common sources of medical images. For instance, CT scans have proven useful in the assessment of pulmonary conditions and have demonstrated sound potential in supporting early diagnosis of COVID-19 [16]. Nevertheless, it requires transfer of the patient to the CT department, a platform-sterilization routine before conducting the test, and the need for the involvement of experts before and after the procedure [17].

X-ray images provide another useful and cost-effective means of computerized detection of COVID-19 and other lung infections [15]. However, as the disease progresses, the image features may become less informative [18,19].

Ultrasound scanning, on the other hand, offers the possibility to transfer the probing device to the patient room with limited infectious implications and less radiation exposure and provides remarkable diagnostic contributions [13].

In this context, medical image analysis has been tailored to the detection of many conditions, such as malaria [20], diabetes [21], glaucoma [22], tuberculosis [23], and many types of cancer [24,25,26], among others [27]. Nevertheless, traditional pipelines remain limited in terms of performance, owing mainly to the rather shallow and often data-specific representation of the image features adopted. Thanks to the advent of powerful processing hardware, deep learning has emerged as a cutting-edge solution in medical applications [28,29,30,31,32,33,34].

On this point, with respect to other medical applications, the assessment of deep architectures for the diagnosis of COVID-19 has not developed a solid literature base of research so far. This may be traced back to (i) the fact that the topic has not matured enough and (ii) the scarcity of representative public datasets. Although much attention has been given to deep learning-based COVID-19 image analyses since the outbreak of the virus, still, there is much room for improvement.

This paper presents a novel deep learning pipeline for automatic analysis of COVID-19 using different types of chest medical data, namely Chest X-ray (CXR) and CT images. The main contributions of this study are as follows:

(1) Unlike previous deep learning frameworks that only used one type of data, this work uses both CXR and CT images.
(2) For the automatic detection and classification of COVID-19, we propose a new approach based on Vision Transformer (ViT).
(3) The development of a Siamese encoder that employs a distillation technique to classify original and augmented images.

The remainder of this paper is organized as follows. Section 2 provides a narrative of existing deep learning work for COVID-19 diagnosis. Section 3 describes the details of the proposed methodology. Section 4 describes the adopted data and the experimental setup. Section 5 presents and discusses the results. Section 5 concludes the paper.

## 2. Related Work

The processing of COVID-19 images aims to determine the existence of features potentially associated with infection, namely unilateral or bilateral ground-glass opacities, distributed peripherally, mostly in round and oval shapes [35,36,37]. A comprehensive review for machine learning techniques used for COVID-19 detection and classification based on CXR or CT images was provided in [38].

Some contributions follow a traditional scheme by combining such features with a classifier to infer the presence of infection. For instance, Mahdy et al. [39] used a multi-level thresholding for segmenting the X-ray images. The segments were then classified using a Support Vector Machine (SVM) classifier. Barstugan [40] first proceeded with SVM-based classification without any feature selection and then with features selected via five feature selection methods. The best score was observed using a grey level size zone matrix feature selector along with SVM classification.

Thus far, the literature has accumulated various deep learning methods for COVID-19 detection in X-ray and CT images. For X-ray images, Marques et al. presented an EffecientNet pipeline to classify chest X-ray images into the classes COVID-19, normal, or pneumonia following 10-fold cross validation [41]. Zabirul Islam et al. combined a convolutional neural network (CNN) and a long short-term memory network for COVID-19 detection in X-ray images [42]. In [43], the authors proposed a multiscale attention-guided deep network with soft distance regularization to detect COVID-19 in X-ray images. The proposed network generated a prediction vector and attention from multiscale feature maps. Furthermore, to render the model more robust and to populate the training data, attention-guided augmentations along with a soft distance regularization were adopted. In [44], wavelet decomposition was incorporated into a convolutional neural network to enable multiresolution analysis. The authors in [45] proposed detecting COVID-19 in X-ray data by implementing several uncertainty estimation methods such as Softmax scores, Monte-Carlo dropout, and deterministic uncertainty quantification. An ensemble of deep learning models was presented in [46], where weighted averaging was applied according to the sensitivity of each model towards each class. Heidari et al. fine-tuned a pre-trained VGG16 model to classify X-ray images into three classes [47]. Abbas et al. applied transfer learning from object recognition (i.e., ImageNet dataset) to X-ray images. The transfer was carried out in three steps, namely (i) decomposition, which consists in applying class decomposition to AlexNet-extracted deep local features; (ii) the transfer phase, where the network weights were fine-tuned for X-ray images; and (iii) the compose phase, which assembles the subclasses of each class [48]. The dependence of these methods on CXR in the diagnosis reduces the sensitivity of the results of early detection because the sensitivity increases with the progression of the disease [18,49,50].

Regarding CT images, Amyar et al. [51] constructed a deep network that consisted of a 10-convolutonal-layer encoder stage, a 9-convolutional-layer decoder part for reconstruction, and a 9-convolutional-layer decoder part for segmentation. Xu et al. implemented a VNet and an inception residual network for feature extraction and region proposal network for region-of-interest segmentation [52]. Sun et al. presented a two-stage feature selection method, namely, a deep forest to learn the high-level features and an adaptive feature selection to find the discriminative features. The selected features were then fed to the four-criteria classifier [53]. Ko et al. also used transfer learning to compare four pre-trained deep convolutional networks and obtained their best result using ResNet-50 [54], while Wu et al. transferred the knowledge of a Res2Net and appended an enhanced feature model to detect COVID-19 cases in a two-class CT dataset [55]. In [56], a CT image synthesis approach based on a conditional generative adversarial network was proposed to deal with data shortage. Horry et al. proposed a noise-reduction pre-processing step to prepare a hybrid dataset of X-ray, CT, and US images, and the data were then fed into a VGG19 network [57]. Although processing CT datasets yields better results when diagnosing COVID-19 [18,58], there will be always restrictions in reducing patients’ exposure to radiation, which limits the availability of a CT dataset that can optimize the performance of model diagnoses alone [59,60].

## 3. Methodology

Let us consider $S={\left\{X_{i},y_{i}\right\}}_{i=1}^{n}$, a set of n chest medical images, where $X_{i\;}\mathrm{and}\;y_{i}\;\;\mathrm{are}$ representative images and their corresponding class labels, $y_{i}\in \left\{1,2,\ldots ,m\right\}$, and m is the number of defined classes for this set.

The aim of the proposed method is to learn mapping from the input chest image to the correct class label. The model is based on a Data-Efficient Image Transformer (DeiT) architecture, which is an improved version of Vision Transformer (ViT). ViT’s architecture is fully based on a Data-Efficient Image Transformer (DeiT) architecture, which is an improved version of Vision Transformer (ViT). The architecture of ViT is based entirely on the vanilla Transformer [61], which has garnered a lot of attention in recent years due to its ability to achieve state-of-the-art (SOTA) performance in machine translation and other natural language-processing applications [62]. The Transformer architecture is made up of encoder–decoder blocks that allow sequential data to be handled in parallel without the use of any recurrent networks. The success of Transformer models largely comes from the self-attention mechanism, which is proposed to capture long-range relationships between the sequence’s elements. Vision Transformer was proposed as an attempt to extend the use of the standard Transformer to image classification. The main goal was to generalize image classification on modalities other than text without integrating any data-specific architecture. In particular, ViT utilizes the encoder module of the Transformer to perform classification by mapping a sequence of image patches to the semantic label. Unlike the conventional CNN architectures, which typically use filters with a local receptive field, the attention mechanism employed by the Vision Transformer allows it to be used over different regions of the image and to integrate information across the entire image.

**Figure 1:**
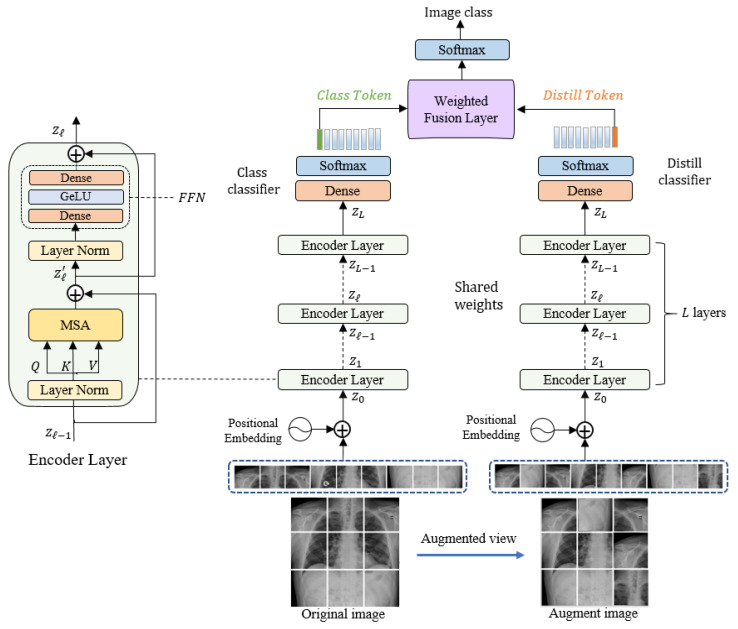
The overall structure of the proposed model.

Our proposed model is composed of three main blocks: an embedding layer, a Siamese encoder, and a decoder. The original input image from the training set is processed to generate an augmented input image; then, these two images (original and augmented) are subdivided into non-overlapping patches and fed into the embedding layer, followed by the Siamese encoder. The encoder is also connected to two independent classifiers: the token and distiller classifiers. In the following subsections, we discuss the model’s components in detail. Figure 1 illustrates the overall structure of the proposed model.

### 3.1. Linear Embeddimg Layer

First, an augmented view image is generated from the original image by applying a data-augmentation technique. These two images are then converted into a sequence of non-overlapping patches. The original input image x and the augmented image of dimension h×w×c (where h, w, and c are the height, width, and number of channels, respectively) are then converted into a sequence of length m by dividing it into small patches $x=\left\{x_{p}^{1},x_{p}^{2},\cdots ,x_{p}^{m}\right\}$ of a fixed dimension of p×p and $m=h\times w/p^{2}$. These patches are analogous to word tokens in the original Transformer. Before feeding the sequence of patches into the encoder, it is linearly projected into a vector of the model dimension $d_{model}$ using a learned embedding matrix E. The embedded representations are then concatenated together along with a learnable class token $x_{class}$ that is required to perform the classification task. The flattened image patches are converted into embeddings by feeding them into a linear embedding layer E to match their dimension to the model dimension $d_{model}$.

To prevent losing the positional information because of the flattening process, each patch embedding is added to its corresponding positional information. The resultant position-aware embeddings are appended with a learnable class token $x_{class}$. Since the decoder is adopted from DeiT architecture, another distillation token $x_{distil}$ is appended along with the class token to the patch embeddings, as shown in Equation (1). The two tokens and the patch embeddings interact with each other via a self-attention mechanism.

(1) $$z_{0}=\left[x_{class};x_{distil};x_{p}^{1}E;x_{p}^{2}E;\ldots ;x_{p}^{m}E\right]+E_{pos}WhileE\in R^{\left(p^{2}.c\right)\times d_{model}},\wedge E_{pos}\in R^{\left(m+2\right)\times d_{model}}\;$$

### 3.2. Siamese Encoder Module

The Siamese architecture of the encoder is adopted from the Data-Efficient Image Transformer (DeiT) architecture. DeiT is an enhanced version of ViT, where less training data are required. The encoder consists of a stack of L identical layers, each one composed of two main blocks: a multi-head self-attention (MSA) block, and a feed-forward network (FFN) block. The MSA, which is a key component of the Transformer encoder, utilizes the self-attention (SA) mechanism to find dependencies between different patches of the input image. Equations (2) and (3) show the details of the calculations that take place in the SA block. First, three different matrices—key K, the query Q, and the value V—are generated from the input sequence using three linear layers. By applying an inner product for matching query matrix against the key matrix, an attention-map is generated. The SoftMax function is applied to obtain the output after scaling it by the dimension of the key $d_{K}$. Finally, the result is multiplied with the value V to focus on more important values.

(2) $$\left[Q,K,V\right]=zU_{QKV};\;U_{QKV}\in R^{d_{model}\times 3d_{K}}$$

(3) $$SA\left(z\right)=softmax\left(QK^{T}/\sqrt{d_{K}}\right).V$$

The multi-head self-attention is an extension of SA, in which it runs the SA process in parallel using multiple self-attention heads ($SA_{1},SA_{2}\ldots SA_{h}$), where h is the number of heads. The aim of using h head is that each head can focus on different relations among the image patches. The outputs of all heads are then concatenated together and projected to the final dimension by a linear layer, as in Equation (4):

(4) $$MSA\left(z\right)=Concat\left(SA_{1}\left(z\right);SA_{2}\left(z\right);\ldots SA_{h}\left(z\right)\right)W^{O},\;W^{O}\in R^{h.d_{K}\times d_{model}}$$

where $W^{O}$ represents the learned parameters of the final projection matrix.

FNN is the second block in the encoder layer that follows the MSA block. It consists of two fully connected layers with a *GeLU* activation function [63] in between. A layer of normalization (LN) proceeds each of the two encoder layer’s blocks. By applying residual connections, the outputs are computed according to the following Equations (5) and (6):

(5) $$z_{l}^{\prime }=MSA\left(ln\left(z_{l-1}\right)\right)+z_{l-1},\;l=1\ldots L\;\;$$

(6) $$\;z_{l}=FNN\left(ln\left(z_{l}^{\prime }\right)\right)+z_{l}^{\prime },\;l=1\ldots L\;$$

Similarly, the encoder receives the augmented view of the image, which is subdivided into a sequence of patches. To generate the second view of the image, we applied different image-augmentation techniques. Data-augmentation techniques are appropriate for increasing the size and diversity of the limited-size training dataset, which is the case for medical images datasets. Several data-augmentation techniques that are based on applying simple geometric transformations such as rotating, cropping, or shifting or applying color transformations such as modifying the brightness or the contrast of the images have been implemented in the literature. Recently, several advanced data-augmentation techniques have been applied in the detection of COVID-19 using medical images on generative adversarial network (GAN) [64,65], conditional generative adversarial networks (CGAN) [66], and AdvProp [67]. More sophisticated techniques based on random erasing and image-mixing have been introduced recently to generate more challenging samples for the model such as the Cutout [68], Mixup [69], and CutMix [70] techniques. In Cutout, a random fixed-size region of the image is intentionally replaced with black pixels or random noise. The process of randomly erasing regions boosts the model to learn from the entire image’s context rather than relying on a specific visual feature. One limitation of using Cutout is losing information since erasing some regions could remove informative parts of image objects [70]. In this paper, we utilize the Cutout technique to generate augmented images from the original images.

### 3.3. Classification Layer

The output of the Siamese encoder is fed into the classification layer, which is composed of two connected classifiers: the class and distiller classifiers. Each one is composed of a fully connected layer (FC) with a SoftMax activation function to determine the class labels. We feed the first element of the encoder output $z_{L}^{0}$, which represents the classification token to the class classifier.

(7) $$y_{class}=Softmax\left(FC\left(z_{L}^{0}\right)\right)$$

The second token $z_{L}^{1}$ represents the distillation and is passed to the distiller classifier.

(8) $$y_{distiller}=Softmax\left(FC\left(z_{L}^{1}\right)\right)$$

Then, the outputs are fed into a weighted average fusion layer followed by SoftMax layer to obtain the final class of the predicted class of the input image according to the following equation:

(9) $$y=\frac{1}{2}(\;y_{class}+y_{distil})$$

### 3.4. Network Optimization

To learn the model for the binary (CT dataset) or multi-class (CXR dataset) classification, we use the following loss function:

(10) $$L\left(x_{ij},y_{ij}\right)=\frac{-1}{n}\overset{w}{\underset{i=1}{\sum }}\overset{h}{\underset{j=1}{\sum }}y_{ij}log\frac{1}{1+e^{-x_{ij}}}+\left(1-y_{ij}\right)log\left(1-\frac{1}{1+e^{-x_{ij}}}\right)$$

where w and h are number of training images and defined classes, respectively; ground-truth labels are represented by $y_{ij}\in {\left\{0,1\right\}}^{c}$ (CT dataset) or $y_{ij}\in {\left\{0,1,2\right\}}^{c}$ (CXR dataset), and $x_{ij}\in \left[0,1\right]$ is the predicted probability. The learning is performed by minimizing a total loss consisting of two terms given by the following equation:

(11) $$L_{total}=L\left(z_{L}^{0},y_{g}\right)+L\left(z_{L}^{1},y_{g}\right)$$

where L represents the binary cross-entropy loss, shown in Equation (10), $y_{g}$ states the ground-truth labels, $z_{L}^{0}$ is classification tokens, and $z_{L}^{1}$ represent the distillation tokens.

In the following Algorithm 1, we provide the main steps for training and testing the model.
**Algorithm 1:** Main steps for training and testing the model.
Input: Training set of n chest images $S={\left\{X_{i},\;Y_{i}\right\}}_{i=1}^{n}$ with corresponding ground-truth labels. Output: test images predicted class labels
1.Set parameters of the model:
Image size: 224.Patch size p: 16.Mini-batch size b: 50.Learning rate: 0.0001.Optimizer: Adam
2.Set the number of mini-batches as: $n_{b}=n/b$3.For iteration = 1: number of iteration (25)3.1*For batch = 1 number of mini batches*Augmented view images.Feed the obtained training set batch to the Siamese encoder’s class branch.Feed the generated batch of augmented images to the Siamese encoder’s distill branch.Classification token is fed to the token classifier and distiller token is fed to distiller classifier.Loss is calculated using Equation (11)Loss Backpropagation.Update the parameters of the model.4.Feed the test images to the model.5.Feed the model with test images6.Calculate the predicting labels using the weighted average fusion of the two outputs $y_{class}\;$ *and* $y_{distiller}$ according to Equation (9).

## 4. Experiments

### 4.1. Datasets Description

In our work, we evaluate the proposed model on two CT and CXR datasets, as detailed below:

#### 4.1.1. Chest X-ray Dataset

**Figure 2:**
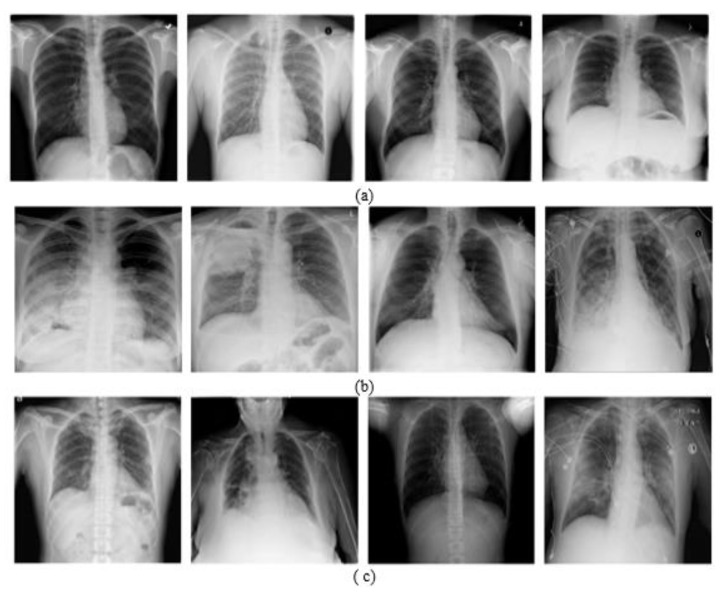
Samples of patients from the COVIDx dataset: (**a**) healthy (normal), (**b**) pneumonia, and (**c**) COVID-19.

The first dataset is a CXR dataset called the COVIDx dataset, proposed by Wang et al. [71]. This dataset was collected from multiple datasets and amounts to CXR images from 13,870 patients. The images were collected and modified from the following data sources: COVID-19 Image Data Collection [72], Figure 2 COVID-19 Chest X-ray Dataset Initiative [73], ActualMed COVID-19 Chest X-ray Dataset Initiative [74], RSNA Pneumonia Detection Challenge dataset [75], and COVID-19 radiography database [76].

COVIDx is the largest open access dataset in terms of the number of positive COVID-19 cases. It is composed of images from three classes, i.e., COVID-19, pneumonia, and normal, and contains 358 CXR images from 266 COVID-19 cases, 8066 normal cases (i.e., no pneumonia), and 5538 cases with non-COVID19 pneumonia. Table 1 shows the number of images per class with the split ratio between the training and testing, where the test part of this dataset was composed of 300 images equally divided between the three classes.

#### 4.1.2. Chest X-ray Dataset

**Figure 3:**
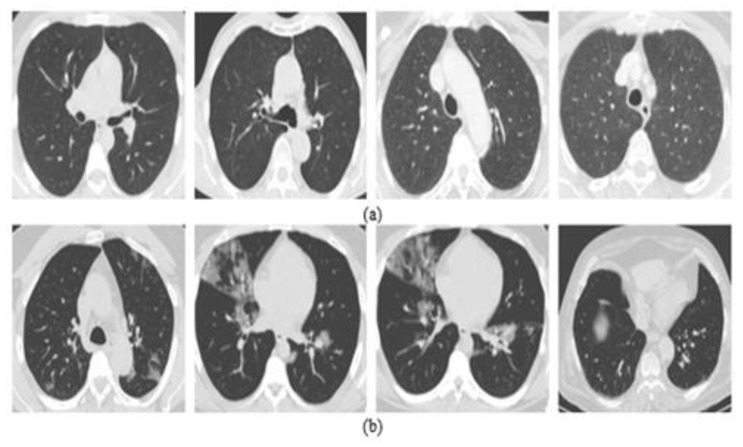
Samples of patients from the CT dataset: (**a**) non-COVID-19 and (**b**) COVID-19.

The second dataset is named the SARS-CoV-2 CT scan dataset, which was collected from hospitals in Sao Paulo, Brazil [77]. It is composed of 2482 CT scan images (1252 CT scan images of 60 patients with COVID-19 infection and 1230 CT scan images of 60 patients without COVID-19 infection). Detailed characteristics of each patient have been omitted by the hospitals due to ethical concerns. Figure 3 depicts some examples of the CT scan images of patients with and without COVID-19 infection.

### 4.2. Evaluation Measures

We followed standard evaluation measures typically adopted in the state-of-the-art [78], yet we report the results in terms of accuracy, precision, recall, specificity, and F-measure (F1 score):

(12) $$\;Accuracy=\frac{TP+TN}{TP+FPz+TN+FN}$$

(13) $$Precision=\frac{TP}{TP+FP}$$

(14) $$\;\;Sensitivity=Recall=\frac{TP}{TP+FN\;}$$

(15) $$Specificity=\frac{TN}{TN+FP\;}$$

(16) $$\;\;F1=2*\;\frac{Precision*Recall}{Precision+Recall}$$

where *TP*, *TN*, *FP*, and *FN* denote true positives, true negatives, false positives, and false negatives, respectively. Accuracy is a common measure of correctly classified (*TP* and *TN*) samples over the total number of samples, as expressed by Equation (12). Precision and recall are often adopted along with accuracy in detection problems. Precision determines how many of the positive predictions are correct, which is calculated by dividing the number of correctly classified positives (*TP*) by the total samples predicted as positives (*TP* and *FP*), as expressed by Equation (13). On the other hand, recall (also known as sensitivity) is calculated by dividing the number of correctly classified positive cases by the total number of all actual positive (*TP* and *FN*) cases, as expressed by Equation (14). It expresses the tendency of a model to identify infected cases [78]. Specificity determines the ability of the model to detect non-infected cases (i.e., similar to recall for positive cases), which is calculated by dividing the number of correctly classified negative cases (*TN*) by the number of all actual negative (*TN* and *FP*) cases, as expressed by Equation (15). Furthermore, F-measure, or F1 score, is considered a balance between precision and recall, which is obtained by calculating the weighted harmonic mean of both precision and recall, as presented in Equation (16).

### 4.3. Experimental Setup

We conduct several experiments, and each experiment was repeated three times. First, we simulated the scenarios of previous state-of-the-art work, where some contributions allocated 60% and others 80% of the dataset for training and where the remainder was set for testing purposes. Then, we reported the results of the proposed model considering a realistic scenario, in which only 20% of the available data was placed for training.

The proposed model was implemented in Pytorch, where we used the AdaBelief optimization algorithm to train the network [79]. The experiments were conducted using a workstation with i9 CPU @ 2.9 GHz, 32 GB of RAM, and NVIDIA GeForce GTX 1080 Ti (11 GB GDDR5X).

## 5. Results

In this section, we present and discuss the results of the experiments to evaluate the proposed pipeline. The average and detailed values of the results are reported and discussed in terms of the aforementioned evaluation measures. First, we present the results on the individual datasets and display the activation maps of the processed images at different layers of the network. Second, we analyze the sensitivity of the model towards different scenarios of the availability of training data. Finally, we compare the results of the model on the described datasets against the SOTA.

### 5.1. Results on CXR

**Figure 4:**
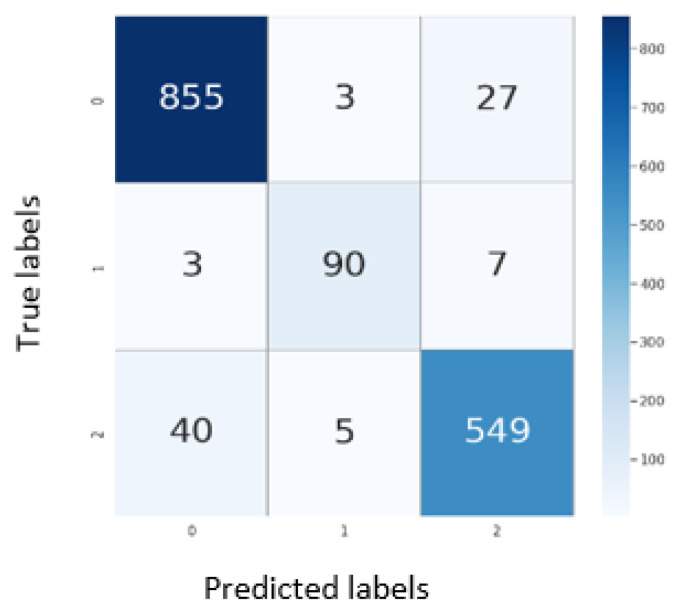
Confusion matrix for the evaluation on test set of COVIDx dataset, where the labels 0, 1, and 2 represents the normal, COVID-19, and pneumonia classes, respectively.

**Figure 5:**
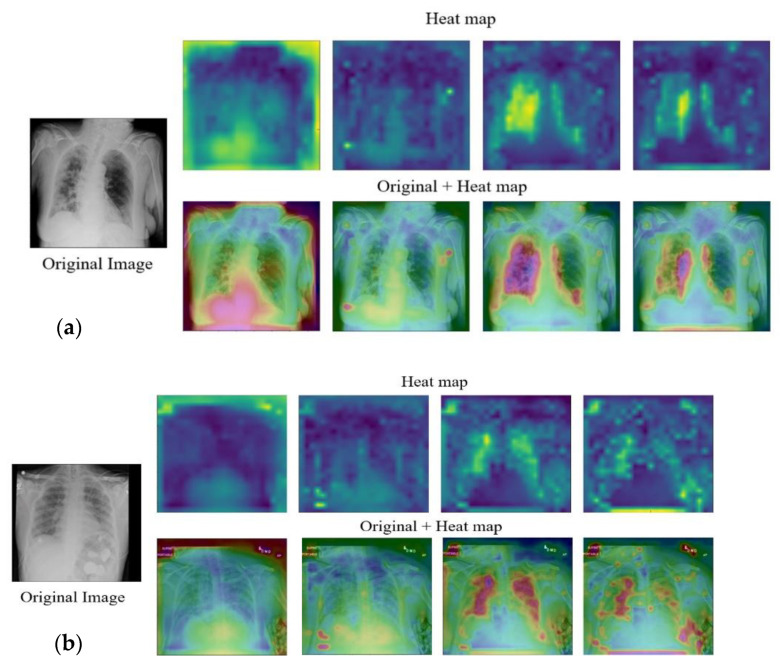
Heat maps of the COVIDx images: (**a**) COVID-19 and (**b**) pneumonia.

In this subsection, we present the results of the proposed model on the COVIDx dataset. Table 2 shows the overall and per-class classification results in terms of accuracy, precision, recall, specificity, and F1 score), while Figure 4 depicts the corresponding confusion matrix. The results indicate that the proposed model exhibits good performance in terms of all of the evaluation measures. The overall accuracy of the model is equal to 94.62%, with an accuracy over 90.0% for each class. The precision, recall, and F1 score of the model amounted to 96.77%, while the overall specificity yield was 99.65%. This confirms the ability of the proposed pipeline to correctly detect positive cases and to discard irrelevant cases. In Figure 5, we show heat maps generated by analyzing X-ray images from different layers of the model. They demonstrated the progression of the focus region over layers. The model appears to focus on random locations in the initial layers. As the image proceeds through the model layers, the network focuses increasingly on regions that have a strong and consistent relationship with the image’s class. Finally, the key zones of the lungs that reflect COVID-19 or pneumonia observations objects are highlighted in the last layer.

### 5.2. Results on CT

Table 3 shows detailed results of the model on the CT dataset, where we used 60% of the dataset for training and 40% for testing. We repeat the experiments three times. The average values exceeded 99.10% across all measures, with a standard deviation of less than 0.50%. These results confirm the power and the stability of the model in classifying COVID-19 cases from CT images.

**Figure 6:**
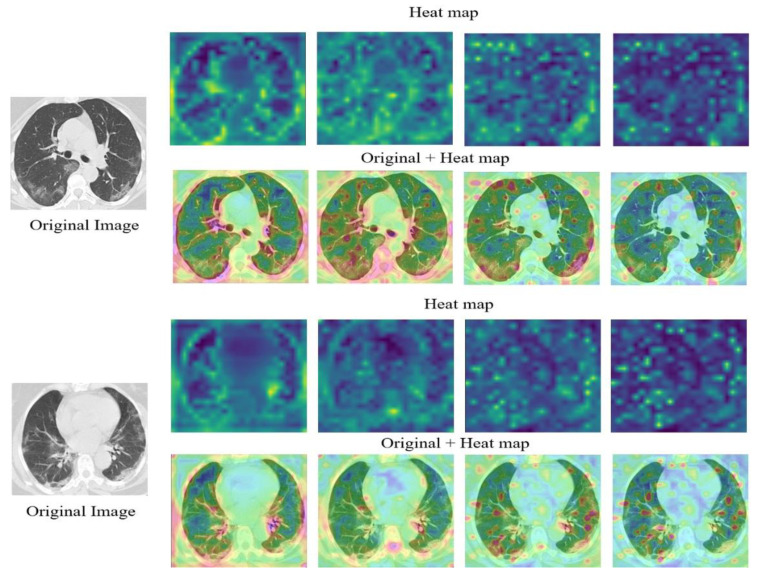
Heat maps of CT images of COVID-19 cases.

On the other hand, in order to assess the robustness of the proposed approach, we split the dataset using different training to-testing ratios. Table 4 shows the average classification accuracies in the case of s 80%:20% split. The average values for accuracy, precision, recall, specificity, and F1 score were 99.13, 99.46, 98.82, 99.47, and 99.13, respectively. Table 5 reports the average results in terms of a 20%:80% split, and the results are proof that, even when we reduce the training size, the accuracies remain comparable with those of the 80%:20% split, suggesting a potential real-time use of the proposed approach. In Figure 6, heat maps derived from the model’s various layers are shown. They show the progression of focus areas over network layers, similar to X-ray images, where the network learns to highlight relevant places consistent with the assessed conditions. The last layer of the network, for example, tends to emphasize zones of the lungs that represent COVID-19 instances, such as bilateral and peripheral ground glass and consolidative pulmonary opacities [80], as illustrated in Figure 1. Table 6 compares the proposed model’s findings with those of SOTA works on the same CT dataset (i.e., the SARS-CoV-2 CT scan dataset).

In terms of all performance indicators, our model clearly outperforms the results of all SOTA works. Our model, in particular, improves the accuracy, precision, recall, and F-measure by 0.64%, 1.31%, 0.04%, and 0.71%, respectively.

## 6. Conclusions

In this study, we proposed a deep learning-based framework for the detection of Coronavirus disease 2019 via two common types of medical images, namely CT and X-ray. The Vision Transformer architecture was used as a backbone to the proposed pipeline, in which a Siamese encoder was applied. The Siamese encoder was developed to process the class token and distillation token. Moreover, we employed atrous convolution at different rates to produce denser features from multi-scale feature maps. To augment the dataset, we generated adversarial examples, which clearly improved the performance. The classification results revealed that our proposed framework outperforms state-of-the art deep learning techniques. The proposed framework has demonstrated its robustness under limited training data. We believe that the proposed architecture potentially suits a multimodal scenario.

## Figures and Tables

**Table 1:** Numbers of images per class in the COVIDX dataset.

	Normal	Pneumonia	COVID-19
Train	66	5438	258
Test	100	100	100
Total	8066	5538	358

**Table 2:** Classification results (expressed as a percent) obtained on the COVIDX dataset.

	Overall	Per Class		
		Normal	COVID-19	Pneumonia
Accuracy	94.62	96.61	90	92.42
Precision	96.77	95.21	92.84	94.17
Recall	96.77	97.61	90	92.42
Specificity	99.65	93.7	99.43	96.53
F1	96.77	95.91	90.91	93.29

**Table 3:** Classification results (expressed as a percent) obtained on the CT dataset with a split of 60:40.

	Trial 1	Trial 2	Trial 3	Avg ± sd.
Accuracy	99.09	99.19	99.59	99.29 ± 0.26
Precision	98.57	99.39	99.41	99.12 ± 0.48
Recall	99.58	98.98	99.8	99.45 ± 0.42
Specificity	98.61	99.39	99.38	99.13 ± 0.45
F1	99.08	99.18	99.68	99.31 ± 0.32

**Table 4:** Classification results (expressed as a percent) obtained on the CT dataset with a split of 80:20.

	Trial 1	Trial 2	Trial 3	Avg ± sd.
Accuracy	99.40	98.99	98.99	99.13 ± 0.23
Precision	98.77	99.60	100.00	99.46 ± 0.63
Recall	100.00	98.41	99.5	98.82 ± 1.04
Specificity	99.82	99.59	100.00	99.47 ± 0.6
F1	99.38	99.00	99.01	99.13 ± 0.22

**Table 5:** Classification results (expressed as a percent) obtained on the CT dataset with a split of 20:80.

	Trial 1	Trial 2	Trial 3	Avg ± sd.
Accuracy	99.55	99.01	99.55	99.37 ± 0.31
Precision	99.6	98.18	99.6	99.13 ± 0.82
Recall	99.5	99.79	99.5	99.6 ± 0.17
Specificity	99.6	98.28	99.6	99.16 ± 0.76
F1	99.55	98.98	99.55	99.36 ± 0.33

**Table 6:** Classification results (expressed as a percent) obtained on the CT dataset with a split of 80:20, 60:40, and 20:80.

	Training-to-Testing Ratio (%)	Accuracy	Precision	Recall	F1
Alrahhal et al. [67]	80:20	99.24	99.16	99.25	99.21
Soares et al. [77]		97.38	99.16	95.53	97.31
Silva et al. [81]		98.99	99.20	98.80	98.99
Proposed		99.13	99.46	98.82	99.13
Alrahhal et al. [67]	60:40	98.65	97.81	99.41	98.60
Pathak et al. [82]		98.37	98.74	98.87	98.14
Proposed		99.29	99.12	99.45	99.31
Alrahhal et al. [67]	20:80	96.16	96.90	95.41	96.15
Proposed		99.37	99.13	99.60	99.36
